# Longitudinal hippocampal volumetric changes in mice following brain infarction

**DOI:** 10.1038/s41598-021-88284-7

**Published:** 2021-05-13

**Authors:** Vanessa H. Brait, David K. Wright, Mohsen Nategh, Alexander Oman, Warda T. Syeda, Charlotte M. Ermine, Katrina R. O’Brien, Emilio Werden, Leonid Churilov, Leigh A. Johnston, Lachlan H. Thompson, Jess Nithianantharajah, Katherine A. Jackman, Amy Brodtmann

**Affiliations:** 1 The Florey Institute of Neuroscience and Mental Health, University of Melbourne, Parkville, VIC Australia; 2 The Department of Neuroscience, Central Clinical School, Monash University, Melbourne, VIC Australia; 3 Melbourne Medical School, University of Melbourne, Parkville, VIC Australia; 4 Department of Biomedical Engineering, University of Melbourne, Parkville, VIC Australia; 5 Melbourne Brain Centre Imaging Unit, University of Melbourne, Parkville, VIC Australia

**Keywords:** Translational research, Stroke, Dementia

## Abstract

Hippocampal atrophy is increasingly described in many neurodegenerative syndromes in humans, including stroke and vascular cognitive impairment. However, the progression of brain volume changes after stroke in rodent models is poorly characterized. We aimed to monitor hippocampal atrophy occurring in mice up to 48-weeks post-stroke. Male C57BL/6J mice were subjected to an intraluminal filament-induced middle cerebral artery occlusion (MCAO). At baseline, 3-days, and 1-, 4-, 12-, 24-, 36- and 48-weeks post-surgery, we measured sensorimotor behavior and hippocampal volumes from T_2_-weighted MRI scans. Hippocampal volume—both ipsilateral and contralateral—increased over the life-span of sham-operated mice. In MCAO-subjected mice, different trajectories of ipsilateral hippocampal volume change were observed dependent on whether the hippocampus contained direct infarction, with a decrease in directly infarcted tissue and an increase in non-infarcted tissue. To further investigate these volume changes, neuronal and glial cell densities were assessed in histological brain sections from the subset of MCAO mice lacking hippocampal infarction. Our findings demonstrate previously uncharacterized changes in hippocampal volume and potentially brain parenchymal cell density up to 48-weeks in both sham- and MCAO-operated mice.

## Introduction

Ischemic stroke remains the second highest cause of mortality and a leading cause of long-term disability worldwide, despite the fact that global incidence and mortality rates have reduced over the last 25 years^1^. Brain atrophy after ischemic stroke is increasingly described in humans, and occurs in areas of direct infarction, as well as in areas that are distant from but still connected to the primary infarct site^2,3^, a process known as secondary neurodegeneration (SND). Clinically, both ischemic stroke and stroke risk factors have been associated with atrophy of the hippocampus^4–10^. In humans, we have previously reported a hippocampal volume reduction of 3.9% after a first-ever stroke and 9.2% after a recurrent stroke at three months. Mean atrophy rates between 3 months and 1 year post-stroke were 0.5% for controls and 1.0% for stroke patients (0.6% contralesionally, 1.4% ipsilesionally)^5^. Importantly, the hippocampus is usually considered a region remote to the infarct, given that direct infarction of the hippocampus is relatively rare^11^, yet it is still observed to undergo SND following functional and structural disconnection from the infarcted site^7,12,13^. Moreover, post-stroke cognitive impairment is often observed in combination with a reduction in hippocampal volume or a deformation of the hippocampus, although other structures are also observed to atrophy^2,4,6,7,10,14–16^. It is possible that ischemic infarction may lead to remote hippocampal injury, further contributing to cognitive impairment^7^.

In rodents, ipsilesional hippocampal atrophy post-stroke has also been observed^9,17–19^. However, few researchers have looked beyond 24-weeks, and the trajectory of post-stroke hippocampal volume change over multiple, extended time points is unknown. Such assessment is critical given that SND is a delayed response to ischemic stroke, often occurring days to weeks after the initial insult^20^. The activation of microglia and astrocytes is a hallmark of post-stroke SND^21–23^, and several authors have suggested the likely involvement of microglia and astrocytes in the SND process^22–25^. While the acute immune response to MCAO-induced ischemia has previously been described^26,27^, the longitudinal trajectory of microglial and astrocytic proliferation and the subsequent pathophysiological SND response to stroke remains poorly understood.

In addition, the natural progression of hippocampal volume changes in the normal rodent brain with advancing age has not been well-described. Whilst brain atrophy is an almost universal part of normal aging^28,29^, it may be related to human life-style risk factors^8^ and may not occur in rodents. Therefore, it is necessary to first understand and depict the brain trajectories of normal rodent aging to establish a baseline that enables more rational interpretation of possible stroke-related changes. Therefore, we aimed to examine the volume of the hippocampus up to 48-weeks post-MCAO in wild-type mice, using sham-operated mice as their controls. We hypothesized that in sham-operated mice hippocampal volume would increase initially due to normal growth, but then remain stable in adulthood, and that ipsilesional hippocampal atrophy would occur post-stroke, regardless of whether the hippocampus was directly infarcted.

## Results

### Mortality, infarct and edema volume and sensorimotor deficit following transient MCAO

**Figure 1:**
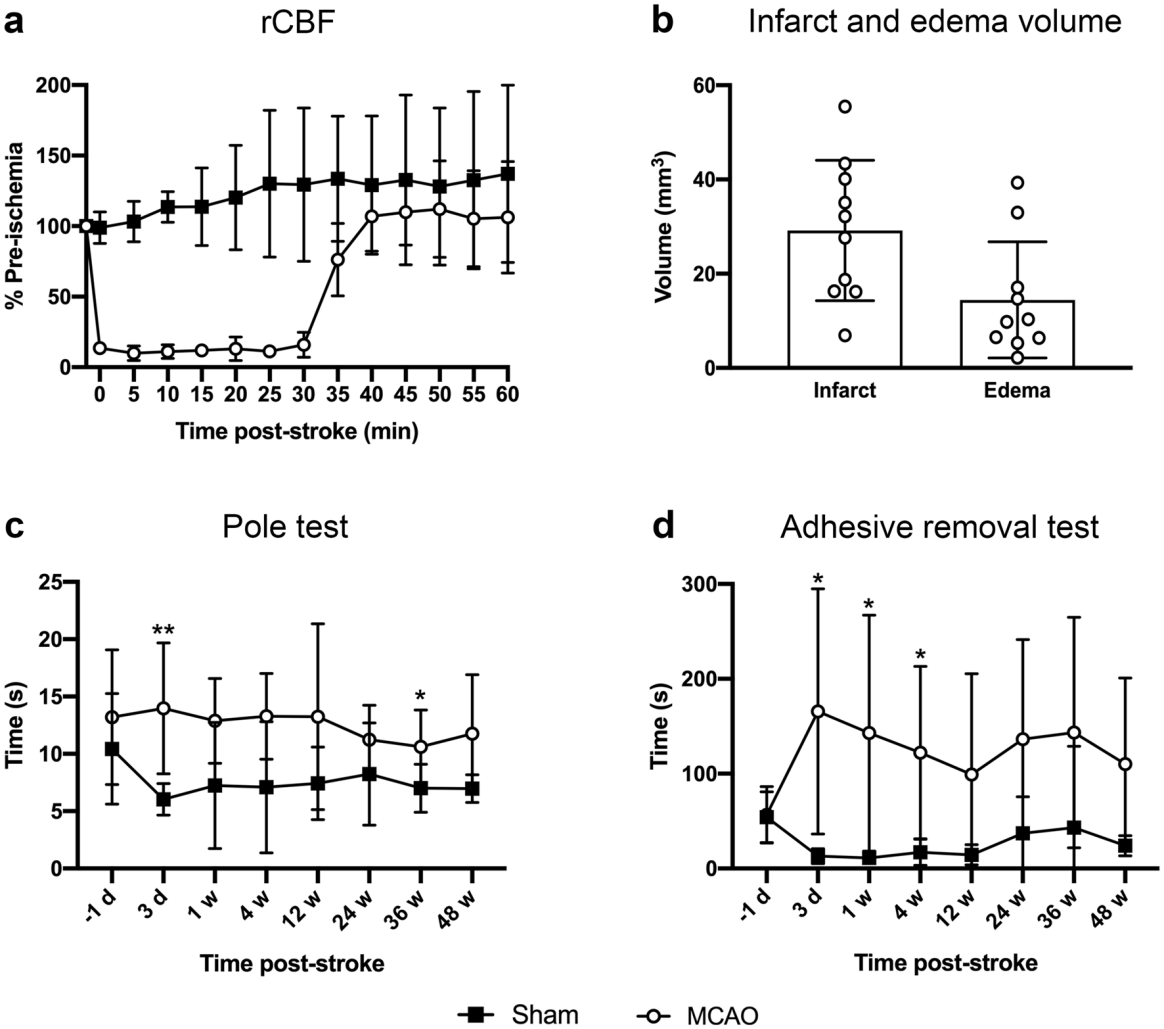
Regional cerebral blood flow (rCBF), infarct and edema volume and sensorimotor tests in mice subjected to MRI. (**a**) Regional cerebral blood flow during sham-surgery (black squares) and MCAO-surgery (white circles; n = 11). (**b**) Infarct and edema volume at 3-days post-MCAO (n = 10). (**c**) Times taken to turn around and descend the pole in sham- and MCAO-operated mice at each time point measured (n = 10–11 for sham and n = 11 for MCAO). (**d**) Times taken to remove the adhesive on the left forepaw in sham- and MCAO-operated mice at each time point measured (n = 10–11 for sham and n = 11 for MCAO). Data are presented as mean ± s.d. *p < 0.05; **p < 0.01, mixed effects analysis followed by a Bonferroni post-hoc test.

Following insertion of the filament, regional cerebral blood flow (rCBF) reduced by > 75% of the pre-ischemia baseline in all mice. During the 30-min ischemic period, rCBF was maintained at an average of 12% of the pre-ischemic baseline, and after filament removal, it returned to baseline (Fig. 1a). Mortality rate following MCAO was 5/49 (10%; 1/12 for MRI cohort and 4/37 for histology cohort); all assisted deaths due to ethical requirements. The 30-min ischemia produced an infarct volume of 29.19 ± 14.91 mm^3^ at day 3, of which 17.75 ± 10.87 mm^3^ was cortical and 11.44 ± 4.42 mm^3^ was subcortical, and an edema volume of 14.44 ± 12.34 mm^3^ (Fig. 1b).

A corresponding deficit in sensorimotor function was demonstrated using the pole test (evaluating motor skills and coordination) and the adhesive removal test (evaluating paw and mouth sensitivity and correct dexterity), and showed impairments in MCAO-operated mice up to 48-weeks post-stroke compared to sham-operated mice. Specifically, mice subjected to MCAO were significantly slower than sham-operated mice at turning around and descending the pole at 3-days (*p* < 0.01) and 36-weeks (*p* < 0.05) post-surgery (Fig. 1c), and were significantly slower at removing the adhesive on their left forepaw at 3-days, 1- and 4-weeks (*p* < 0.05) post-surgery (Fig. 1d).

### Longitudinal hippocampal volume increases in sham-operated mice

**Figure 2:**
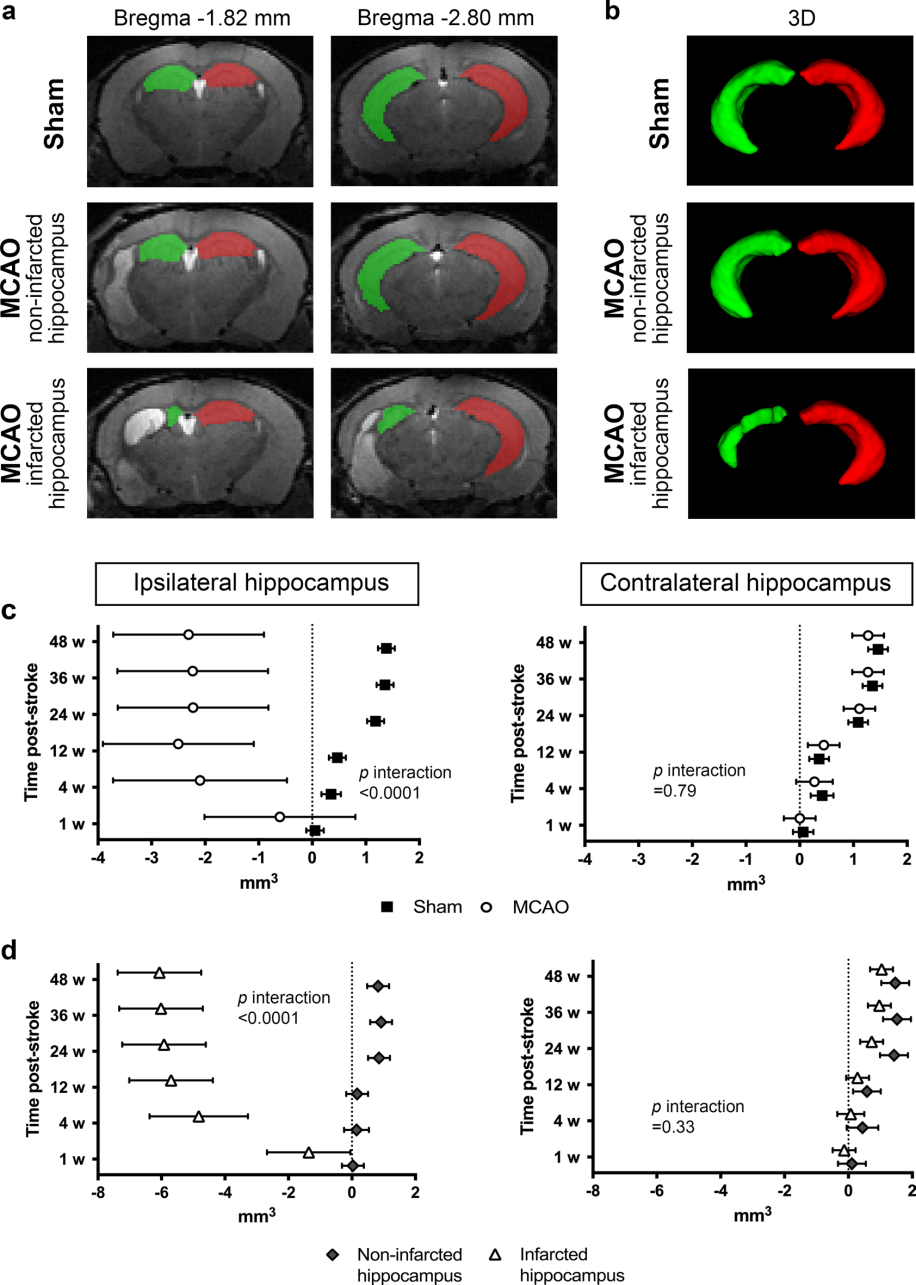
Hippocampal volume. (**a**) Representative T_2_-weighted images of a sham-operated mouse, an MCAO-operated mouse with a non-infarcted hippocampus, and an MCAO-operated mouse with an infarcted hippocampus with manual segmentation of the ipsilateral (green) and contralateral (red) hippocampus at 12-weeks post-surgery. (**b**) Representative 3D images of a sham-operated mouse, and an MCAO-operated mouse with and without an infarcted hippocampus with manual segmentation of the ipsilateral (green) and contralateral (red) hippocampus at 12-weeks post-surgery. (**c**) Volume changes of the ipsilateral and contralateral hippocampus in sham- (black squares) and MCAO-operated mice (white circles) at each time point measured (n = 7–11). (**d**) The MCAO-operated mice were further subdivided into those with and without an infarcted hippocampus. Volume changes of the ipsilateral and contralateral hippocampus in MCAO-operated mice with a non-infarcted hippocampus (gray diamonds) and with an infarcted hippocampus (white triangles) at each time point measured (n = 3–6). Random effect generalized least squares regression test. The effect sizes are presented as mean differences in volume (mm^3^) between the given time point and baseline with corresponding 95% confidence intervals (*p* < 0.05 when the error bars do not touch the dotted line at 0, and the further the deviation from this, the greater the difference).

Using T_2_-weighted (T2*w*) MRI, we were first interested to characterize hippocampal volume changes over time in control (sham-operated) mice. As expected, ipsilateral (right) and contralateral (left) hippocampal volumes were similar at baseline (6 weeks of age—10.57 ± 0.39 mm^3^ and 10.79 ± 0.34 mm^3^, respectively). Similarly, no significant volume change between the right and left hippocampal volume were observed at 1-week post-sham surgery (7 weeks of age). By 4 weeks, a significant, proportional increase in volume was observed in both hemispheres (of 2.81 ± 0.51% and 3.33 ± 0.42%, respectively; 10 weeks of age), which further increased at 24-weeks post-surgery (of 11.29 ± 1.22% and 10.15 ± 1.37%, respectively; 30 weeks of age), and was maintained at 36- and 48-weeks post-sham surgery (42 and 54 weeks of age, respectively) (Fig. 2c). All *p*-values are shown in Supplementary Table S1 online.

### Longitudinal hippocampal volume changes in MCAO-operated mice

We next examined hippocampal volume changes over time in MCAO-operated mice, regardless of the presence or absence of direct hippocampal injury. As expected, the ipsilateral and contralateral hippocampal volumes were similar at baseline—10.58 ± 0.56 mm^3^ and 10.68 ± 0.47 mm^3^, respectively. In the ipsilateral hippocampus, no significant volume change was observed from baseline to 1-week post-stroke. However, a reduction was observed at 4-weeks post-stroke which was maintained at 12-, 24-, 36- and 48-weeks post-stroke (between 18.14 and 24.29% smaller; Fig. 2c). In the contralateral hippocampus, there was no significant difference in volume at either 1- or 4-weeks post-stroke compared to baseline. Interestingly, we observed a modest increase in contralateral hippocampal volume at 12-weeks (4.16 ± 1.04%), with a further increase observed at all time points measured from 24-weeks post-stroke (between 10.38 and 11.89% larger than baseline; Fig. 2c)—a similar pattern to that observed in the sham-operated mice. All *p*-values are shown in Supplementary Table S1 online.

### Differences in hippocampal volume between sham- and MCAO-operated mice

**Figure 3:**
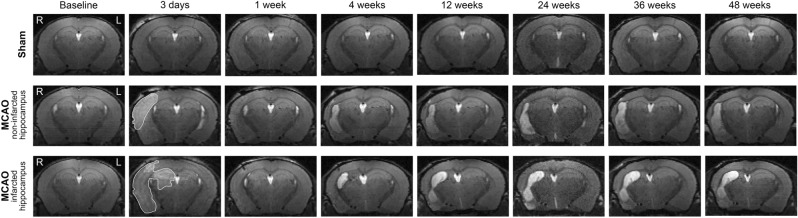
Representative T_2_-weighted images of a sham-operated mouse, an MCAO-operated mouse with a non-infarcted hippocampus, and an MCAO-operated mouse with an infarcted hippocampus. R (right) is the ipsilateral hemisphere and L (left) is the contralateral hemisphere, and all images have the same orientation. The hyperintense region in the ipsilateral hemisphere at 3-days post-stroke (area outlined in white) is the infarcted tissue.

The average changes in ipsilateral hippocampal volume over time were significantly different between MCAO- and sham-operated mice (*p* < 0.0001; Fig. 2c), whereas no difference in the contralateral hippocampal volume was found (*p* = 0.79; Fig. 2c). Representative T2*w* images with hippocampal segmentation are shown in Fig. 2a, and 3D images of the hippocampal segmentation are shown in Fig. 2b. Representative T2*w* images of a sham-operated mouse, and of an MCAO-operated mouse with and without an infarcted hippocampus at each time point are shown in Fig. 3.

### Exploring the impact of direct hippocampal injury

In order to establish whether the atrophy observed in the ipsilateral hippocampus was due to the direct infarction of the hippocampus, or whether it could be considered secondary atrophy occurring remote to the infarct site, the MCAO-operated mice were subdivided into those with (n = 5) and without (n = 6) direct hippocampal infarction at 3-days post-stroke. The presence or absence of direct hippocampal injury had no influence on changes in contralateral hippocampal volume (*p* = 0.33 Fig. 2d), whereas ipsilateral hippocampal volume over time was significantly different between the two groups (*p* < 0.0001; Fig. 2d), suggesting that the stroke lesion itself does not produce remote hippocampal atrophy in mice. In the mice without hippocampal infarction, ipsilateral hippocampal volume remained constant relative to their baseline at 1-, 4- or 12-weeks post-stroke, but was significantly larger at later time points (24-, 36- and 48-weeks post-stroke; between 7.60 and 8.53% larger than baseline; Fig. 2d). In contrast, mice with hippocampal infarction had a significantly smaller ipsilateral hippocampal volume compared to baseline at all time points measured (between 13.12 and 58.96% smaller than baseline; Fig. 2d). The profile of volume changes in the contralateral hippocampi of MCAO-operated mice with or without direct hippocampal infarction were similar to that found in the contralateral hippocampus of sham- and MCAO-operated mice (Fig. 2c vs. 2d; all *p*-values are shown in Supplementary Table S1 online).

### Longitudinal volume changes are observed in both dorsal and ventral regions of the hippocampus after MCAO

**Figure 4:**
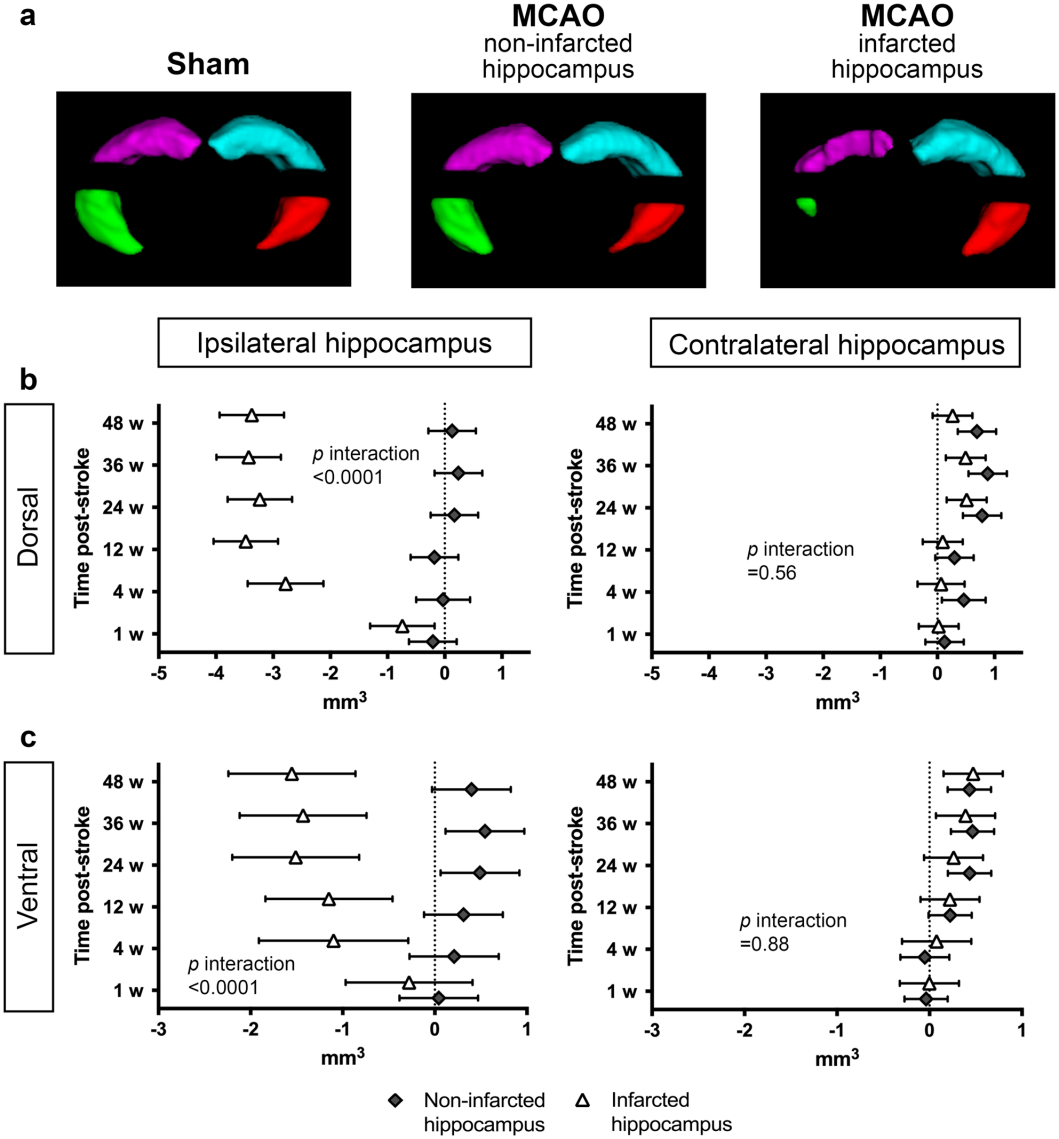
Dorsal and Ventral Hippocampal Volumes. (**a**) Representative 3D images of a sham-operated mouse, an MCAO-operated mouse with a non-infarcted hippocampus, and an MCAO-operated mouse with an infarcted hippocampus with automatic segmentation of the dorsal ipsilateral (purple), dorsal contralateral (aqua), ventral ipsilateral (green) and ventral contralateral (red) hippocampi at 12-weeks post-surgery. Volume changes of the dorsal (**b**) and ventral (**c**) ipsilateral and contralateral hippocampus in MCAO-operated mice with a non-infarcted hippocampus (gray diamonds) and with an infarcted hippocampus (white triangles) at each time point measured (n = 3–6). Random effect generalized least squares regression test. The effect sizes are presented as mean differences in volume (mm^3^) between the given time point and baseline with corresponding 95% confidence intervals (*p* < 0.05 when the error bars do not touch the dotted line at 0, and the further the deviation from this, the greater the difference).

To establish whether the reduction in hippocampal volumes in mice were region specifc, we divided the hippocampal segmentations into dorsal and ventral sections (Fig. 4). The dorsal and ventral hippocampal volumes at baseline are shown in Table 1.

**Table 1:** Dorsal and ventral hippocampal volumes (mm^3^) at baseline (mean ± s.d.).

	Sham	MCAO non-infarcted hippocampus	MCAO infarcted hippocampus
Right dorsal hippocampus	5.94 ± 0.42	6.03 ± 0.34	5.73 ± 0.46
Left dorsal hippocampus	6.00 ± 0.19	6.29 ± 0.20	6.12 ± 0.33
Right ventral hippocampus	2.56 ± 0.27	2.63 ± 0.26	2.63 ± 0.28
Left ventral hippocampus	2.64 ± 0.23	2.43 ± 0.26	2.44 ± 0.15

The average changes in dorsal and ventral ipsilateral hippocampal volume over time were significantly different between MCAO-operated mice with and without an infarcted hippocampus (*p* < 0.0001), although a similar reduction was observed in both these regions (a 60% reduction by 48-weeks), suggesting the effect was not region specific (Fig. 4b, c). There was no difference in volume of either the dorsal or ventral contralateral hippocampus between MCAO-operated mice with and without an infarcted hippocampus (*p* = 0.56 and *p* = 0.88, respectively; Fig. 4b, c), and the profile of volume change was similar to that found in the entire contralateral hippocampus (Fig. 2d vs. Fig. 4b, c; all *p*-values are shown in Supplementary Table S2 online). These findings again support that the MCAO does not affect the volume of remote hippocampi.

### Histological estimation of hippocampal neuronal, microglial, and astrocytic density in sham- versus MCAO-operated mice

To further explore the observed changes in volume, we performed a limited histological assessment. Immunohistochemistry was performed at all time points from 1-week post-surgery. Mice with direct hippocampal infarction were removed from quantitative analysis (n = 2), so that only hippocampi remote from the ischemic infarct were analyzed. The animal numbers for each antibody and time point are shown in Supplementary Table S3 online. It is important to note that although the majority of groups have 4 to 6 animals, a third have only 2 or 3 animals, and therefore these findings must be interpreted with caution.

To confirm a similar degree of volume changes in mice studied for histology and those studied for MRI, we analyzed the correlation in total hippocampal hemisphere volume. As shown in Table 2, the Pearson’s correlation coefficients show good agreement between the mean hippocampal volume changes measured over time in the 12-week-old mice used for histology and in the 6-week-old mice used for the MRI (only including those without an infarcted hippocampus) in all groups except in the contralateral hippocampi of MCAO-operated mice, validating the trend of an increasing volume. Significance was reached in the ipsilateral hippocampi of both sham- and MCAO-operated mice (*p* < 0.05). The animal numbers for the volume measurements are shown in Supplementary Table S4 online.

**Table 2:** Pearson’s correlation coefficient test.

	Sham		MCAO	
	Ipsilateral	Contralateral	Ipsilateral	Contralateral
Pearson’s correlation coefficient	0.86	0.70	0.88	0.57
*p*-value	0.03*	0.12	0.02*	0.23

*p < 0.05.

**Figure 5:**
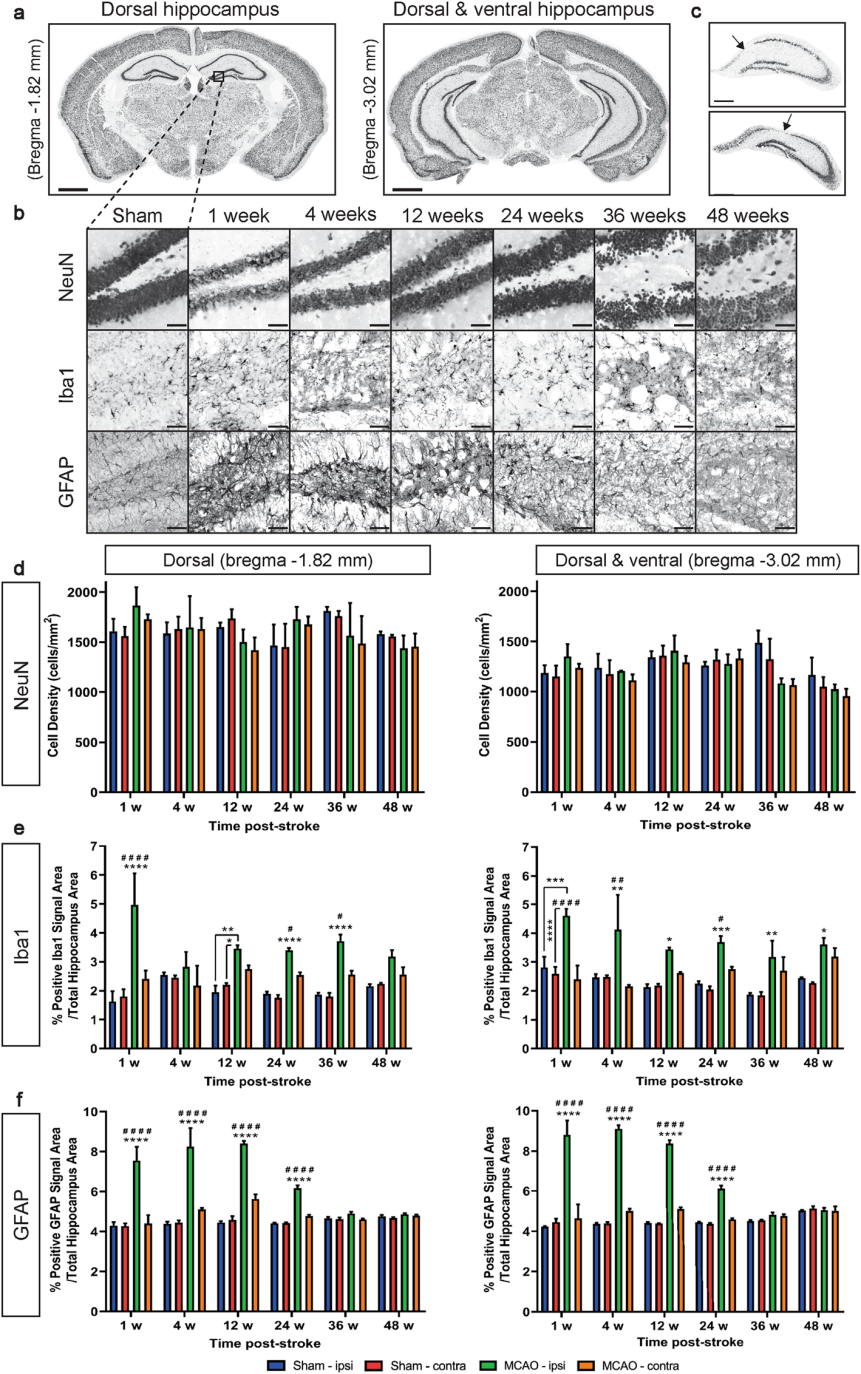
Histological Data. (**a**) A representative coronal image of sections -1.82 mm and -3.02 mm relative to bregma. Scale bar: 1 mm. (**b**) Representative NeuN (neurons), Iba1 (microglia), and GFAP (astrocytes) staining in the hippocampus of a sham-operated mouse, and an MCAO-operated mouse at each time point at bregma -1.82 mm. Scale bar: 50 µm. (**c**) Representative images of CA1 neurodegeneration in the ipsilateral hippocampus of an MCAO-operated mouse at 12-weeks (top image) and 24-weeks post-stroke (bottom image). Arrow indicates neurodegeneration. Scale bar: 250 µm. Neuronal (**d**), microglial (**e**) and astrocytic (**f**) density in the ipsilateral and contralateral hippocampus at bregma −1.82 mm and −3.02 mm in sham-operated mice (blue and red, respectively) and MCAO-operated mice (green and orange, respectively) at each time point measured (n = 2–6). Data are presented as mean ± s.e.m. *p < 0.05; **p < 0.01; ***p < 0.001; ****p < 0.0001 compared to both ipsilateral and contralateral sham unless otherwise specified, ^#^*p* < 0.05; ^##^*p* < 0.01; ^####^*p* < 0.0001 compared to contralateral MCAO, two-way ANOVA followed by a Sidak post-hoc test.

Neuronal density in the hippocampus, assessed using NeuN, was performed to examine changes in the number of neurons. Although no hippocampal atrophy was observed, some of these brains exhibited neurodegeneration in the CA1 section of the ipsilateral hippocampus (representative images of the CA1 neurodegeneration are shown in Fig. 5c). This included one mouse sacrificed at 1-week, two mice at 12-weeks and one mouse at 24-weeks post-stroke. Group data showed no significant differences between neuronal density in sham- or MCAO-operated mice at any of the time points measured, or within groups across the different time points, in either of the sections—1.82 mm (dorsal hippocampus) and 3.02 mm caudal to bregma (dorsal and ventral hippocampi) (Fig. 5d). The hippocampal volume of the brains presenting with CA1 degeneration were not consistently smaller than their contralateral hippocampi (data not shown). A representative image of the two matched coronal sections is shown in Fig. 5a, and representative NeuN^+^ cells in the hippocampus of a sham-operated mouse, and an MCAO-operated mouse at each time point, are shown in Fig. 5b.

We next performed immunohistochemistry targeting microglia (Iba1) and astrocytes (GFAP), as major cellular components of the inflammatory response. Representative Iba1^+^ and GFAP^+^ staining is shown in Fig. 5b. In the rostral sections encompassing the dorsal hippocampus, there was a significant interaction between time point and treatment (type of surgery and hemisphere; *p* = 0.0008). We found a significantly higher Iba1^+^ density in the ipsilateral hippocampus of MCAO-operated mice, compared to the ipsilateral and contralateral hippocampi of sham-operated mice, at 1-, 12-, 24- and 36-weeks post-stroke (Fig. 5e). There was also a significant difference between the ipsilateral and the contralateral hippocampi of MCAO-operated mice at 1-, 24- and 36- weeks (Fig. 5e). In caudal sections including dorsal and ventral hippocampi, there was a significant treatment (type of surgery and hemisphere) and time effect, but no interaction. We also found a significantly higher Iba1^+^ density in the ipsilateral hippocampus of MCAO-operated mice, compared to the ipsilateral and contralateral hippocampi of sham-operated mice, at all time points measured (Fig. 5e). A significant difference between the ipsilateral and contralateral hippocampi of MCAO-operated mice was also observed at 1-, 4-, and 24-weeks post-stroke (Fig. 5e).

The density of astrocytes in the hippocampus were also affected by MCAO. In both the rostral and caudal sections of the hippocampus, there was a significant interaction between time point and treatment (type of surgery and hemisphere; *p* < 0.0001). Furthermore, there was a significantly higher GFAP^+^ density in the ipsilateral hippocampus of MCAO-operated mice compared to the ipsilateral and contralateral hippocampus of sham-operated mice, as well as compared to the contralateral hippocampus of MCAO-operated mice, at 1-, 4-, 12- and 24-weeks post-stroke (*p* < 0.0001). Pronounced peaks were observed at 1-, 4- and 12-weeks, and a resolution of the astrocyte response to MCAO was seen by 36-weeks post-stroke (Fig. 5f).

## Discussion

We examined changes in hippocampal volume in sham- and MCAO-operated mice up to 48-weeks post-surgery (6-to-54 weeks of age). In MCAO-operated mice, we found significant atrophy in the ipsilateral hippocampus from 4-weeks post-stroke, but only when the hippocampus was directly infarcted. Atrophy was not observed in the absence of direct injury. These data support atrophy occurring as a result of local injury rather than as a secondary, remote process. This aligns with what has previously been found in rodent stroke studies, where ipsilesional hippocampal atrophy was observed in animals with an infarct in the hippocampus^18,19^ or where the presence of hippocampal infarction was not clarified^9,17^. In order to further examine this atrophy and determine if it was region specific, we assessed changes in functionally distinct areas of the hippocampus—the dorsal region, predominately implicated in cognitive processes such as learning and memory, and the ventral region, more associated with motivation and emotions^30^. We observed a similar decrease in the volume of both the dorsal and ventral ipsilateral hippocampi of MCAO-operated mice with a hippocampal infarct, suggesting that both cognitive and emotional processing pathways can be affected by stroke. This is consistent with clinical data reporting a high prevalence of post-stroke mood disorders in patients^31^ and may indicate similar impacts in rodents. In future studies it will be important to correlate these findings with longitudinal cognitive testing. Importantly, we were able to demonstrate pronounced and sustained deficits in sensorimotor function in our studies within the limits of these tests, providing validation of the model.

As we did not find atrophy in the absence of direct injury, our volumetric findings differ somewhat to studies of hippocampal volume in clinical stroke populations, where ischemic stroke was found to be associated with accelerated hippocampal atrophy without hippocampal infarction^4,6,7,9,10^. There are a number of possible explanations for these differences. Firstly, hippocampal vulnerability may pre-date the stroke due to the impact of cerebrovascular disease. In the CANVAS study, a cohort study of patients with ischemic stroke and healthy age- and sex-matched controls^8^, Werden and colleagues showed that brain volume may already be compromised at the time of the stroke. People with vascular risk factors, such as type 2 diabetes, hypertension and atrial fibrillation, are found to have hippocampal atrophy^32–34^, a finding that has been recapitulated in rodent models of type 2 diabetes and hypertension^35,36^. Furthermore, new evidence suggests that vascular risk factors in mid-life are most strongly associated with lower brain volume in later life^37^. These data strongly suggest that chronic mid-life disease processes are involved in causing both the stroke itself, and the accelerated structural brain aging, therefore suggesting that a cumulative vascular burden causes brain atrophy, which leads to cognitive deficits. Thus, our results are perhaps not surprising given that mice used in our study were otherwise healthy and not suffering co-morbidities. This may speak to the need to model vascular risk factors and co-morbidities to increase the clinical translation in stroke models. Another possibility is that the brains of adolescent mice are more plastic and prone to regenerative pathways and are perhaps therefore better able to compensate for these injuries. However, we did not see hippocampal atrophy in the adult histological cohort either, suggesting that this is unlikely to be the reason.

In the sham-operated mice, there was a progressive increase in hippocampal volume bilaterally from 10 to 30 weeks of age, demonstrating that hippocampi continue to increase in size with increased age. There was also no change in hippocampal volume from 30 to 54 weeks of age, suggesting that hippocampal atrophy does not occur in normal aging in the mouse, at least up to 54 weeks of age. Whilst on initial inspection this may appear surprising, it likely reflects the paucity of knowledge about brain volume changes in normal rodent aging. Hippocampal volume increases between 5 and 35 weeks of age^38^ and 5 and 12 months of age^39^ in wild-type mice have been described in different degenerative disease models, including Huntington’s disease and frontotemporal lobe degeneration tauopathy. This increase is presumably due to brain maturation in healthy animals. In the unaffected, contralateral hippocampus of MCAO-operated mice, an increase in volume was observed, starting from 24-weeks post-stroke. Taken alone, this larger volume could be due to increased neurogenesis following stroke injury^40^, however, this increase mirrors that observed in sham-operated mice suggesting it may reflect normal maturation and growth of the brain. Another study similarly reported increased volume in the contralateral hippocampus at 6-months (ie 24 weeks) in both sham and MCAO-treated rats^14^, suggesting that this effect is reproducible and that the MCAO does not affect normal hippocampal growth in the contralateral hemisphere. Similar to the contralateral hippocampus, MCAO mice without direct hippocampal injury also had increased ipsilateral volume suggesting that the stroke was not exerting secondary, degenerative effects on either the contralesional hippocampus or the ipsilesional hippocampus when not infarcted. This does not mean however, that neuronal cell death in the hippocampus remote from the infarct site is not occurring, as neurogenesis or immune cell activation or infiltration may be counteracting the volume effects of neuronal cell death.

To address this question in part, we performed limited immunohistochemical examination of the cellular changes in response to MCAO. In the histological cohort, only 6% of mice had a hippocampal infarct, compared to 45% in the MRI cohort. This difference is most likely due to the physical size of the brain of 6-week-old mice compared to 12-week-old mice, and the use of the same size filament (both length and thickness), making it more likely that that the hippocampus, an area outside the MCA territory, becomes infarcted^41,42^. However, in our histological studies, we only analyzed hippocampi remote from the ischemic infarct so mice with direct hippocampal infarction were removed from quantitative analysis. Analysis of neuronal density in both the ipsilateral and contralateral hippocampus revealed similar values between sham- and MCAO-operated groups at all time points measured, suggesting a proportional change in neuronal cell number with volume change, although given the low animal numbers, this needs to be interpreted with caution. It is interesting to note that in selected MCAO-operated mice, neurodegeneration was found in the CA1 section of the ipsilateral hippocampus, the area most vulnerable to neurodegeneration from ischemia^43^. These brains had lower neuronal density, but they did not significantly affect the group data. This shows that there was in fact some SND occurring in selected mice, but because there was no observable pattern in the brains in which this occurred, it may suggest natural variability in infarct volume and within-strain anatomical and genetic variance^44^. The reduction in neuronal cells in the CA1 did not consistently reduce the volume of those hippocampi, suggesting that this may have also been occurring in some mice in the MRI cohort. Furthermore, the volume may not have reduced due to an increase in microglial and astrocyte activation, which may have compensated for neuronal changes in volume.

The current study also provides a longitudinal assessment of microglial and astrocytic activation within the non-infarcted hippocampus up to 48-weeks post-stroke. We show that following MCAO, microglial density is increased up to 48-weeks, whereas astrocytes are increased initially, but return to baseline by 36-weeks, demonstrating that glial activity is increased long after the ischemic insult, even though the hippocampus itself is not infarcted. Ischemic stroke is well-known to activate both microglia and astrocytes, but while the microglial and astrocytic activation in and around the ischemic core in the acute phase of ischemia has been well described^26,27,45,46^, this is the first study to examine long-term expression in the hippocampus, remote from the ischemic lesion, up to 48-weeks post stroke, and it supports a global response of the brain to injury. Microglia and astrocytes can exert both neurotoxic and neuroprotective effects after a stroke, although the exact role is likely to be time-, region- and context-dependent^47–51^, demonstrating that the glial response to ischemic stroke is incredibly complex. Other studies examining glial expression in areas of SND have found an increase in activated microglia and reactive astrocytes in the thalamus at 16-days^52^ and 4-weeks post-stroke^23^, however, it was unclear whether activated glial cells are exerting neurodegenerative or neuroprotective actions at sites of SND^52^. The fact that astrocytic activation returns to baseline by 36-weeks post-stroke, unlike microglial activation which remains high, is an intriguing finding and may reflect the temporal sequence of post-infarction inflammatory changes^53^. Given our animal numbers were relatively small for this sort of analysis, future studies are required to more comprehensively examine the effects of activated microglia and astrocytes in other structures of the brain and areas of SND long-term and to characterize the timeline of glial cell activation exerting protective and deleterious effects.

## Conclusion

We demonstrate novel temporal hippocampal volume changes up to 48-weeks in both sham- and MCAO-operated mice. Hippocampal volume increased over the life-span of sham-operated wild-type mice, due to an increase in the number of neurons. Different trajectories of ipsilesional hippocampal volume change were observed in MCAO-operated mice dependent on whether the stroke lesion involved the hippocampus directly. We did not observe remote hippocampal atrophy as atrophy only occurred with direct hippocampal infarct. Changes in microglial and astrocytic density within the ipsilateral hippocampus of MCAO-operated mice were observed.

## Methods

### Animals

All procedures performed in this study involving animals were approved by the Florey Institute of Neuroscience and Mental Health animal ethics committee. All methods were carried out in accordance with applicable guidelines and regulations for the care and use of animals, and the study was carried out in compliance with the ARRIVE guidelines. Male C57BL/6J mice (n = 90) were purchased from the Animal Resources Centre, Western Australia. All mice were group-housed in individual ventilation cages under standard laboratory conditions including automatically controlled temperature and humidity, a 12-h light/dark cycle and were fed standard laboratory chow and tap water ad libitum. They were acclimatised for a period of 7–8 days. Six-week-old adolescent animals were selected for the MRI study (n = 23), as it was not known whether the animals would live the full 48 weeks if older animals were infarcted. However, we had excellent survival rates for these animals, and so an older cohort (12-week-old adults; n = 67), which models human adult stroke more closely, was included for the histological experiments.

### Focal cerebral ischemia

Transient acute focal cerebral ischemia was induced by intraluminal filament-induced middle cerebral artery occlusion (MCAO), as described previously^54^. Briefly, mice were deeply anesthetized with 5% isoflurane in medical-grade compressed air plus 0.1L/min O_2_ using an anesthetic vaporizer (Harvard Apparatus, Holliston, MA, USA). The level of isoflurane was then reduced and maintained at 1.5%. After dissection of the right common carotid artery, external carotid artery (ECA) and internal carotid artery (ICA), a 6–0 monofilament with silicone-coated tip (602156; Doccol Co., Redlands, CA, USA) was inserted via the ECA into the ICA. This was advanced ~ 9–10 mm past the carotid bifurcation until a sudden drop in regional cerebral blood flow was observed via a fiber optic laser Doppler probe (Moor instruments, Devon, UK), attached to the surface of the cranium at 2 mm posterior and 5 mm lateral to bregma. The filament was kept in place for 30 min, and then removed for reperfusion. Rectal temperature was monitored and maintained at 37.5 ± 0.5 °C throughout the procedure using an electronic temperature controller linked to a heat mat (Coherent Scientific, Hilton, SA, Australia). Sham-operated mice were anesthetized, and the carotid arteries exposed but no filament inserted.

### Sensorimotor tests

MCAO-induced motor deficits were assessed at 8 time points (-1 day (Baseline), 3-days, 1-, 4-, 12-, 24-, 36- and 48-weeks post-surgery) using the pole test and the adhesive removal test. For the pole test, the mice were trained on 2 consecutive days before surgery. On the first day (surgery -2 days), each mouse was placed in a plastic container with the pole in the middle (a 50 cm tall, 1 cm in diameter roughened pole attached to a stable weighted base). After leaving the mouse for a few minutes, it was placed on the top of the pole facing down. Once they descended and touched the ground, they were left to explore the container for 10 s and were then placed on the top of the pole another 4 times. The same procedure occurred on the second day of training (surgery -1 day), except that the mouse was positioned facing up on the top of the pole, and the total time to turn and touch the ground was recorded 5 times, with 10 s between each trial. Testing involved the same process as the second day of training, except that the mouse was only tested 3 times, and an average of these trials was recorded.

For the adhesive removal test, the mice were trained one day before surgery. Training first involved placing the mouse in an empty clear plastic container for 1 min. The mouse was then removed, and a rectangular piece of adhesive tape (3 × 4 mm^2^) was applied to each forepaw using a spatula. The mouse was then returned to the container and the time taken to contact the right and left adhesive with the mouth, and to remove the right and left adhesive were recorded. This was then repeated 4 times (the average was the baseline measurement). Testing involved the same process except that the mouse was only tested once at each time point. Both tests were performed in a quiet environment.

### Animal preparation for MRI

For MRI, mice were anesthetized with 5% isoflurane in a 1:1 mixture of medical-grade compressed air and O_2_ using an anesthetic vaporizer. The mouse was positioned prone on a purpose-built cradle with tooth and ear bars for fixing the head. Anesthesia was maintained using 1–2% isoflurane via a nose-cone and their eyes were protected with Poly Visc lubricating eye ointment (Alcon, Frenchs Forest, NSW, Australia). A small air balloon was placed under the chest to monitor respiration via a pressure transducer. The mice were kept warm with a hot water circulation system built into the cradle. Physiological monitoring of the ventilation rate was continuous. After scanning, mice were returned to warm cages until they woke up. MRI was carried out at the same 8 time points as the sensorimotor testing. At 3-days, only 10 MCAO-operated mice completed the scan; one mouse was excluded due to shallow respiration during MRI. Furthermore, at 4-weeks, four mice from each group were not scanned due to a problem with the RF coil (n = 7).

### MRI data acquisition

MRI was performed using a 4.7 T MRI with Avance III console and cryogenically-cooled transmit/receive surface coil (Bruker, USA) as described previously^55^. A T_2_-weighted image (T2*w*) was acquired using a 2D Rapid Acquisition with Relaxation Enhancement (RARE) sequence with the following parameters: repetition time = 12 s; effective echo time = 40 ms; RARE factor = 8; number of excitations = 4; matrix = 160 × 160; field of view = 19.2 × 19.2 mm^2^; number of slices = 64 with slice thickness = 0.12 mm; and resolution = 0.12 × 0.12 × 0.12 mm^3^.

### MRI analysis

For volumetric analysis, the ipsilesional and contralesional hippocampi were manually traced on ~ 30 consecutive T2*w* image slices encompassing the entire hippocampus, using ITK-SNAP software (http://www.itksnap.org)^56^. Hippocampal segmentation was performed in the coronal plane view. The boundary of the hippocampus was chosen by visually comparing the T2*w* images with the coronal section figures in The Mouse Brain in Stereotaxic Coordinates^57^. Furthermore, to keep the tissue boundaries as consistent as possible across animals, tracing was performed with the previous animal’s segmentation open in a second window. Measurements of hippocampal volume included infarcted tissue (hyperintense regions). After segmentation, the hippocampal volume was calculated based on the number of segmented voxels.

The hippocampus was automatically segmented into fifths according to the height of the contralesional side of the hippocampus measured from the manual segmentation. The dorsal and ventral zones were defined as the two-fifths of the hippocampus from top and bottom, respectively, with an intermediate zone being defined by the middle fifth (similar to that described^30^). A watershed segmentation^58^ was applied to the zone masks, to prevent dorsal-intermediate overlap at the medial tip of the hippocampus. The volume of each segmented region was calculated based on voxel count.

Infarct volume was measured from T2*w* images by manually tracing the hyperintense regions on each slice using ITK-SNAP software. Infarct volume was corrected for edema according to the following formula: CIV = [LHA-(RHA-RIA)] x (thickness of section + distance between sections); in which CIV is corrected infarct volume, LHA is left hemisphere area, RHA is right hemisphere area, and RIA is right hemisphere infarct area^59^. Cortical and subcortical infarcts were separately calculated, with reference to the relevant region boundaries^57^.

### Tissue preparation for histological processing

For histological studies, a separate cohort of mice underwent the same MCAO or sham surgery at 12 weeks of age and were sacrificed at 1-, 4-, 12-, 24-, 36- and 48-weeks post-surgery. After an overdose of Isoflurane, mice were transcardially perfused with cold 0.1 M phosphate buffer solution (PBS; 320 mM Na_2_HPO_4_ anhydrous, 90 mM NaH_2_ PO_4_.H_2_O, pH 7.4) followed by 4% paraformaldehyde (PFA; w/v in 0.1 M PBS, pH 7.4). Brains were removed and post-fixed in 4% PFA overnight at 4 °C. Brains were cryoprotected with 30% sucrose, frozen with dry ice and stored at −80 °C until sectioned. Twenty-micron sections were prepared in a 1:12 series using a cryostat (CM1950, Leica Biosystems, Vic., Australia) and thaw-mounted on Superfrost Plus Adhesion microscope slides (ThermoFisher Scientific, Waltham, MA, USA). Sections were left to dry at room temperature overnight and then stored at −80 °C.

### Immunohistochemistry

Chromogenic immunohistochemistry was performed on slide-mounted sections. Frozen sections were thawed by incubating at 37 °C for 15 min. A hydrophobic barrier was created surrounding each series of sections using an ImmEdge hydrophobic barrier PAP pen (Vector Laboratories, Burlingame, CA, USA). Sections were incubated overnight in the following primary antibodies: rabbit anti-Neuronal nuclear protein IgG (NeuN; Abcam, ab104225, 1:1500), rabbit anti-Ionized calcium binding adaptor molecule 1 IgG (Iba1; Wako, 019–19,741, 1:1500) or rabbit anti-Glial fibrillary acidic protein IgG (GFAP; Abcam, ab7260, 1:1500). Sections targeting NeuN underwent heat-induced antigen retrieval (15 min in 95 °C citrate buffer, pH 6.0) prior to primary incubation. Primary antibodies were detected by incubation with biotin-conjugated Donkey anti-Rabbit IgG secondary antibody (Jackson ImmunoResearch, 711-065-152, 1:400), followed by streptavidin–horseradish peroxidase complex (ABC elite kit, Vectastain) and ultimately exposed to di-amino-benzydine (DAB; Sigma-Aldrich, D8001). Sections were then rinsed three times in PBS (5 min each) and dehydrated in 50%, 70%, 95% and 100% ethanol solutions (30 s each). Finally, sections were cover slipped using DPX Mountant (Sigma-Aldrich) and left to dry at room temperature overnight.

### Image analysis

Chromogenic images were captured using a Pannoramic SCAN II digital slide scanner (3DHISTECH Ltd., Budapest, Hungary) with a 20 × Plan-Apochromat objective (NA 0.8; Carl Zeiss, NSW, Australia). Captured images were viewed by QuPath software^60^ and hippocampal sections were analyzed with FIJI (ImageJ)^61^. Whole ipsilesional and contralesional hippocampal areas were calculated by manually delineating the hippocampal area from 9 consecutive sections across the rostro-caudal extent of the hippocampus (from bregma −1.34 mm to −3.26 mm). To estimate the volume, the area in each NeuN-stained section was multiplied by the distance between sections. For the NeuN, Iba1 and GFAP analysis, two matched coronal sections were selected from the rostro-caudal extent of the hippocampus. Sections were matched via visual inspection of key structural landmarks, guided by the histological images in The Mouse Brain in Stereotaxic Coordinates^57^ (approximately −1.82 mm relative to bregma representing the dorsal zone, and −3.02 mm relative to bregma representing a section with both dorsal and ventral zones). NeuN-positive cells were quantified using the Trainable Weka Segmentation plugin^62^. By training the software to identify a positive neuron, cell counts could be obtained from structures of densely packed neurons typical to the neuroanatomy of the hippocampus. Neuronal density was then determined by dividing the NeuN-positive cell counts by the hippocampal area of that same section. Standardized densities of microglia and astroyctes were calculated by measuring the area of Iba1- and GFAP-immunoreactive cells as a percentage of the hippocampal area of that section.

Only brains lacking hippocampal infarction were analyzed. As such, two MCAO-operated mice (one sacrificed at 12-weeks and one at 48-weeks) were excluded from histological analysis. Other animals were excluded due to a loss of brain tissue integrity through the process of perfusion and tissue handling.

### Statistics

Statistical analyses were carried out using Stata^63^ and GraphPad Prism version 8.0.0 (GraphPad Software Inc., San Diego, CA, USA). Infarct, edema and baseline hippocampal volumes, and rCBF and sensorimotor data are presented as mean ± s.d., and % changes in hippocampal volumes and histological data are presented as mean ± s.e.m. Surgery and time effects for the sensorimotor data were compared using a mixed effects analysis followed by a Bonferroni post-hoc test. The relationship between hippocampal volumes, groups, and time were investigated using random effect generalized least squares regression, with an individual animal as a random effect. To investigate time-by-group interactions, respective interaction terms were included in the regression models. The effect sizes are presented as mean differences in volume (mm^3^) between the given time point and baseline, with corresponding 95% confidence intervals. The strength of the association between the mean change in hippocampal volumes over time using histological versus MRI methods was tested using a Pearson’s correlation coefficient. Surgery and time effects for the histological data were compared using a Two-way ANOVA followed by a Sidak post-hoc test. *p* < 0.05 was considered indicative of statistical significance.

## Supplementary Information

Supplementary Information
