# Supplementary material for: Longitudinal hippocampal volumetric changes in mice following brain infarction
Source: Sci Rep. 2021 May 13;11:10269. doi: 10.1038/s41598-021-88284-7 (PMC8119705; doi:10.1038/s41598-021-88284-7)
Supplement: Supplementary file 1 — Supplementary Information [file 41598_2021_88284_MOESM1_ESM.pdf]

**SUPPLEMENTARY INFORMATION**

# Longitudinal hippocampal volumetric changes in mice following brain infarction

Vanessa H. Brait, PhD, David K. Wright, PhD, Mohsen Nategh, PhD, Alexander Oman, MSc, Warda T. Syeda, PhD, Charlotte M. Ermine, PhD, Katrina R. O'Brien, BSc, Emilio Werden, PhD, Leonid Churilov, PhD, Leigh A. Johnston, PhD, Lachlan H. Thompson, PhD, Jess Nithianantharajah, PhD, Katherine A. Jackman, PhD, Amy Brodtmann, PhD

**Corresponding authors:**
Vanessa H. Brait
The Florey Institute of Neuroscience and Mental Health
University of Melbourne
Parkville, VIC, Australia
Email: vanessa.brait@gmail.com

Amy Brodtmann
The Florey Institute of Neuroscience and Mental Health
University of Melbourne
Parkville, VIC, Australia
Email: agbrod@unimelb.edu.au

**Supplementary Table S1: Ipsilateral and contralateral total hippocampal volume**: *p*-values for comparison between baseline volume and post-surgical time points in sham- and MCAO-operated mice.

| ***p*-values** | **Sham** | | **MCAO** | | **Non-infarcted hippocampus** | | **Infarcted hippocampus** | |
|---|---|---|---|---|---|---|---|---|
| | **Ipsilateral** | **Contralateral** | **Ipsilateral** | **Contralateral** | **Ipsilateral** | **Contralateral** | **Ipsilateral** | **Contralateral** |
| 1-week | 0.532 | 0.499 | 0.401 | 1.000 | 0.874 | 0.621 | 0.043 | 0.469 |
| 4-weeks | <0.0001 | <0.0001 | 0.011 | 0.112 | 0.466 | 0.080 | <0.0001 | 0.734 |
| 12-weeks | <0.0001 | <0.0001 | <0.0001 | 0.003 | 0.354 | 0.009 | <0.0001 | 0.116 |
| 24-weeks | <0.0001 | <0.0001 | 0.002 | <0.0001 | <0.0001 | <0.0001 | <0.0001 | <0.0001 |
| 36-weeks | <0.0001 | <0.0001 | 0.002 | <0.0001 | <0.0001 | <0.0001 | <0.0001 | <0.0001 |
| 48-weeks | <0.0001 | <0.0001 | 0.001 | <0.0001 | <0.0001 | <0.0001 | <0.0001 | <0.0001 |

**Supplementary Table S2: Dorsal and ventral ipsilateral and contralateral hippocampi**: *p*-values for comparison between baseline volume and post-surgical time points in sham- and MCAO-operated mice.

| ***p*-values** | **Non-infarcted hippocampus** | | | | **Infarcted hippocampus** | | | |
|---|---|---|---|---|---|---|---|---|
| | **Dorsal** | | **Ventral** | | **Dorsal** | | **Ventral** | |
| | **Ipsilateral** | **Contralateral** | **Ipsilateral** | **Contralateral** | **Ipsilateral** | **Contralateral** | **Ipsilateral** | **Contralateral** |
| 1-week | 0.330 | 0.462 | 0.843 | 0.758 | 0.010 | 0.897 | 0.428 | 0.992 |
| 4-weeks | 0.907 | 0.018 | 0.394 | 0.708 | <0.0001 | 0.756 | 0.008 | 0.684 |
| 12-weeks | 0.396 | 0.082 | 0.153 | 0.062 | <0.0001 | 0.598 | 0.001 | 0.173 |
| 24-weeks | 0.424 | <0.0001 | 0.024 | <0.0001 | <0.0001 | 0.004 | <0.0001 | 0.110 |
| 36-weeks | 0.260 | <0.0001 | 0.013 | <0.0001 | <0.0001 | 0.005 | <0.0001 | 0.017 |
| 48-weeks | 0.543 | <0.0001 | 0.068 | <0.0001 | <0.0001 | 0.137 | <0.0001 | 0.004 |

**Supplementary Table S3: Animal numbers for the immunohistochemistry.**

| n-values | | Bregma -1.82 | | | | Bregma -3.02 | | | |
|---|---|---|---|---|---|---|---|---|---|
| | | Sham | | MCAO | | Sham | | MCAO | |
| | | Ipsilateral | Contralateral | Ipsilateral | Contralateral | Ipsilateral | Contralateral | Ipsilateral | Contralateral |
| NeuN | 1-week | 4 | 4 | 3 | 3 | 4 | 4 | 4 | 3 |
| | 4-weeks | 5 | 5 | 2 | 2 | 4 | 5 | 2 | 2 |
| | 12-weeks | 5 | 5 | 5 | 5 | 5 | 5 | 5 | 4 |
| | 24-weeks | 4 | 4 | 6 | 6 | 4 | 4 | 5 | 4 |
| | 36-weeks | 4 | 4 | 3 | 3 | 4 | 4 | 4 | 4 |
| | 48-weeks | 5 | 5 | 4 | 4 | 4 | 4 | 4 | 4 |
| Iba1 | 1-week | 3 | 3 | 3 | 3 | 3 | 3 | 4 | 3 |
| | 4-weeks | 5 | 5 | 2 | 2 | 5 | 5 | 2 | 2 |
| | 12-weeks | 5 | 5 | 2 | 2 | 5 | 5 | 2 | 2 |
| | 24-weeks | 4 | 4 | 6 | 6 | 4 | 3 | 5 | 5 |
| | 36-weeks | 3 | 3 | 3 | 3 | 3 | 3 | 4 | 4 |
| | 48-weeks | 3 | 3 | 3 | 3 | 3 | 3 | 3 | 3 |
| GFAP | 1-week | 2 | 2 | 4 | 4 | 2 | 2 | 5 | 4 |
| | 4-weeks | 5 | 5 | 2 | 2 | 5 | 5 | 3 | 3 |
| | 12-weeks | 5 | 5 | 5 | 5 | 5 | 5 | 5 | 5 |
| | 24-weeks | 5 | 5 | 6 | 6 | 5 | 5 | 5 | 6 |
| | 36-weeks | 5 | 5 | 4 | 4 | 5 | 5 | 5 | 5 |
| | 48-weeks | 5 | 5 | 4 | 4 | 5 | 4 | 4 | 4 |

**Supplementary Table S4: Animal numbers for the hippocampal volume calculated by histology.**

| n-values | Sham | | MCAO | |
|---|---|---|---|---|
| | Ipsilateral | Contralateral | Ipsilateral | Contralateral |
| 1-week | 5 | 3 | 4 | 3 |
| 4-weeks | 5 | 5 | 2 | 2 |
| 12-weeks | 5 | 5 | 5 | 5 |
| 24-weeks | 4 | 4 | 6 | 6 |
| 36-weeks | 5 | 5 | 3 | 3 |
| 48-weeks | 5 | 5 | 4 | 4 |
